# Diversity and Distribution of Symbiodinium Associated with Seven Common Coral Species in the Chagos Archipelago, Central Indian Ocean

**DOI:** 10.1371/journal.pone.0035836

**Published:** 2012-05-02

**Authors:** Sung-Yin Yang, Shashank Keshavmurthy, David Obura, Charles R. C. Sheppard, Shakil Visram, Chaolun Allen Chen

**Affiliations:** 1 Biodiversity Research Center, Academia Sinica, Nangang, Taipei, Taiwan; 2 Coastal Ocean Research and Development Indian Ocean (CORDIO), Mombasa, Kenya; 3 School of Life Sciences, University of Warwick, Coventry, United Kingdom; 4 Institute of Oceanography, National Taiwan University, Taipei, Taiwan; University of Texas, United States of America

## Abstract

The Chagos Archipelago designated as a no-take marine protected area in 2010, lying about 500 km south of the Maldives in the Indian Ocean, has a high conservation priority, particularly because of its fast recovery from the ocean-wide massive coral mortality following the 1998 coral bleaching event. The aims of this study were to examine *Symbiodinium* diversity and distribution associated with scleractinian corals in five atolls of the Chagos Archipelago, spread over 10,000 km ^2^. *Symbiodinium* clade diversity in 262 samples of seven common coral species, *Acropora muricata, Isopora palifera, Pocillopora damicornis, P. verrucosa, P. eydouxi, Seriatopora hystrix,* and *Stylophora pistillata* were determined using PCR-SSCP of the ribosomal internal transcribed spacer 1 (ITS1), PCR-DDGE of ITS2, and phylogenetic analyses. The results indicated that *Symbiodinium* in clade C were the dominant symbiont group in the seven coral species. Our analysis revealed types of *Symbiodinium* clade C specific to coral species. Types C1 and C3 (with C3z and C3i variants) were dominant in Acroporidae and C1 and C1c were the dominant types in Pocilloporidae. We also found 2 novel ITS2 types in *S. hystrix* and 1 novel ITS2 type of *Symbiodinium* in *A. muricata*. Some colonies of *A. muricata* and *I. palifera* were also associated with *Symbiodinium* A1. These results suggest that corals in the Chagos Archipelago host different assemblages of *Symbiodinium* types then their conspecifics from other locations in the Indian Ocean; and that future research will show whether these patterns in *Symbiodinium* genotypes may be due to local adaptation to specific conditions in the Chagos.

## Introduction

Mutualistic symbiosis between scleractinian corals and dinoflagellates (genus *Symbiodinium*) contributes to high productivity in coral reef ecosystems, providing important resources and functions for consumers, including humans [1]–[3]. Corals and coral reefs have suffered as a result of several environmental and anthropogenic factors that have caused their destruction worldwide [2], [4]–[10]. Most coral-algal symbioses are sensitive to increasing seawater temperature and high irradiance [2], [11]–[16]. However, particular partner combinations may resist episodes of high thermal stress. Despite the general conclusion that coral-algal mutualistic symbioses are sensitive to changes in their environment, biogeographic studies indicate that different regional environments also significantly influence the ecology and evolution of this relationship [17]–[22]. These environmental factors include average annual temperatures, seasonal changes in water clarity and substantial seawater temperature variation across tropical and subtropical regions [23]–[24].

From various studies, the diversity of *Symbiodinium* is well established [1], [5], [25]–[33]. Molecular (small ribosomal subunit (SSU rDNA), larger subunit (LSU rRNA), chloroplast large subunit ribosomal DNA, internal-transcribed regions (ITS1 and ITS2)) and phylogenetic classification of *Symbiodinium* into functionally distinct evolutionary entities (using alpha-numeric designations equivalent to ‘species’) has shown them to belong to nine divergent phylogenetic ‘clades’ (A to I). [1], [5], [25]–[33]. LaJeunesse [27], [34] using internal transcribed spacer (ITS) region argued that the ‘clades’ of *Symbiodinium* required further resolution into operational taxonomic units, ‘subclade,’ and/or sub species levels, thereby providing a more complete picture of *Symbiodinium* diversity and systematics [34], also see [22] [35]. Furthermore, the results from ITS2 and other analysis now support the designation of the ITS2 sequence as individual *Symbiodinium* species and hence the term ITS2 types is being used to refer to the variation in the *Symbiodinium* diversity [32]. Ecophysiological function has been inferred for several of these clades based either on biogeographic / ecological distribution or physiological stress experiments [7], [17], [22], [35]–[44]. *Symbiodinium* are genetically and physiologically diverse and the presence of a particular *Symbiodinium* type influences a reef coral's tolerance to thermal stress (See [3] for detailed analysis, Also see [20] for *Symbiodinium* evolution and symbiosis response to climate change). Studies have shown that corals associate with different *Symbiodinium* clades or types depending on the environmental conditions.

**Figure 1:**
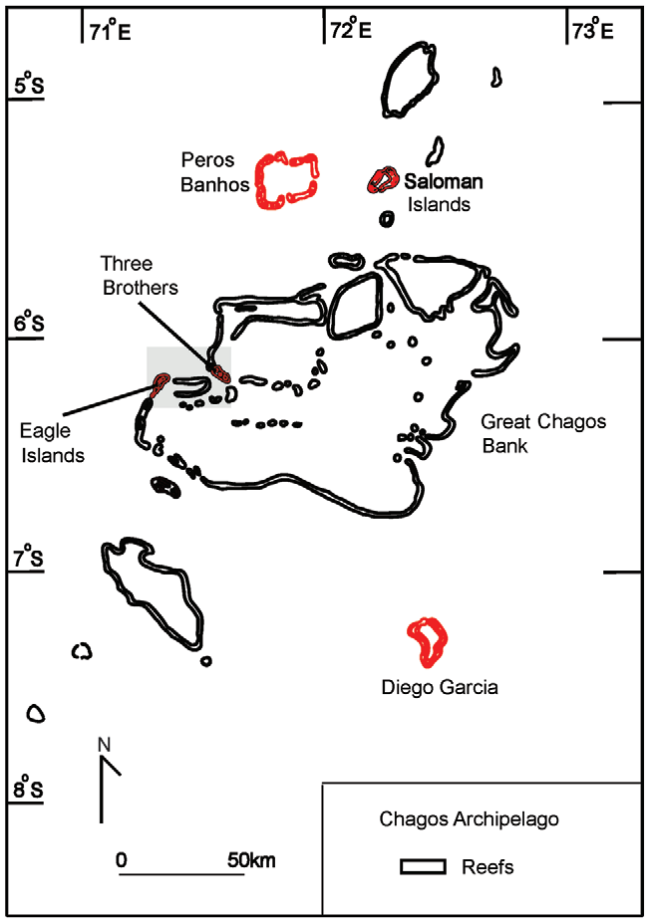
Map showing the location of the Chagos Archipelago, including the four sampling sites: Salomon Islands, Diego Garcia, Chagos Bank and Peros Banhos. Areas shown in red are the actual locations from which the samples were obtained at each site.

**Table 1:** Information on sampling locations, coral species sampled at each location and associated *Symbiodinium* clades (RFLP).

Site	Coral species	28S-RFLP
Salomon Islands (90)	*I. palifera* (17)	C
	*P. damicornis* (24)	C
	*P. eydouxi* (10)	C
	*P. verrucosa* (12)	C
	*S. hystrix* (4)	C
	*S. pistillata* (23)	C
		
Peros Banhos (23)	*A. muricata* (20)	C
	*I. palifera* (3)	C
		
Diego Gracia (78)	*I. palifera* (21)	C (4), C+A (7)
	*P. damicornis* (11)	C
	*S. hystrix* (21)	C
	*S. pistillata* (25)	C
		
Chagos (71)	*A. muricata* (22)	C (11), A (2), C+A (9)
	*I. palifera* (12)	C
	*P. damicornis* (8)	C
	*S. hystrix* (6)	C
	*S. pistillata* (23	C

The numbers in the brackets denote sample obtained from each site, for each coral species and number of clade C and A.

**Table 2:** SSCP and DDGE *Symbiodinium* types associated with each species from different locations.

Coral species	Sample location	SSCP-ITS1	DGGE-ITS2
*A. muricata*	Great Chagos Bank	A1	A1
	Peros Banhos	C	C1
		Ca2	C3z
		Ca3	C3i
		Not detected	C3kk
			
*I. palifera*	Great Chagos Bank	A1	A1
	Salomon Islands	C	C1
	Diego Gracia	Ca	C3
	Peros Banhos	Ca2	C3z
		Ca3	C3i
		Ca4	C40
		Ca5	Not detected
			
*P. damicornis*	Great Chagos Bank	C	C1
	Salomon Islands	C2	C1c
	Diego Gracia	Not detected	C1
			
*P. eydouxi*	Salomon Islands	C	C1
		C2	C1c
			
*P. verrucosa*	Salomon Islands	C	C1
		C2	C1c
			
*S. pistillata*	Great Chagos Bank	C	C1
	Salomon Islands	Cs	Not detected
	Deigo Gracia		
			
*S. hystrix*	Great Chagos Bank	C	C1
	Salomon Islands	Cs	Not detected
	Diego Gracia	Csh	Not detected
		Not detected	C132a, C132

**Figure 2:**
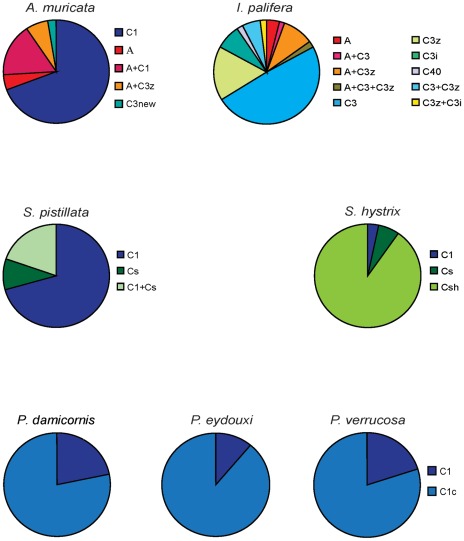
Symbiodinium clade composition in each species. The total sample size for each species is: A. muricata, n = 42; I. palifera, n = 53; S. pistillata, n = 71; S. hystrix, n = 31; P. damicornis, n = 43; P. eydouxi, n = 10; P. verrucosa, n = 12.

Overall composition of Symbodinium clades in the seven coral species sampled from all four locations in the Chagos Archipelago revealed the presence of only two Symbiodinium clades; C and A (Fig. 2 and Fig. S1).

Among the divergent lineages of *Symbiodinium*, clade C is ecologically abundant and diverse among reef corals in both the Indo-Pacific and western Atlantic, whereas clade A is relatively common in shallow water scleractinians in the Caribbean and Indian Ocean [22], [27], [34] but is rare in the Pacific (reviewed in [5], [45], but see [46]). Although surveys have shown that clade C is the dominant symbiont associated with scleractinian corals in the Pacific Ocean and West Indian Ocean [1], [7], [19], [22], [47]–[48], a recent study on the same sampling scale in the north-eastern Indian Ocean (Andaman Sea) showed a significant proportion of clade D and a diversity of *Symbiodinium* types in different coral hosts [22]. Although some regions of the Indian Ocean have been surveyed [7], [22], [49], there is no information of *Symbiodinium* diversity from the central Indian Ocean either at regional or local scale, which limits our understanding of the relationship between coral host and *Symbiodinium* on a broad biogeographical scale.

The Chagos Archiphelago, designated as a no-take marine protected area in 2010 [50]–[51], is located in the central Indian Ocean on the Chagos-Laccadive Ridge that extends as far south as 20^°^S. It covers 550,000 km^2^ with >60,000 km^2^ shallow limestone platform and reefs [51]. Chagos is a valuable location for studying corals, as it contains >25% of the Indian Ocean reef area [51] in a condition and habitat that is largely unaffected by direct, local human impacts, although during the El Niño event of 1998, the Chagos suffered severe coral bleaching and mass mortality especially among large colonies of tabular *Acropora* [52]. However, significant and rapid recovery was observed afterwards [24]. Also, coral diseases are extremely low in the Chagos reefs [51]. The role of the Chagos Archiphelago in the Indian Ocean is very important for a number of reasons, including conservation of commercial fish stocks [50] and supports densities of coral reef fishes one to two orders of magnitude greater than in other Indian Ocean locations [51]. It also provides a scientific reference point for several aspects of Indian Ocean research and for global studies of reef condition and reef restoration. Further, the Chagos archipelago appears to act as a marine biodiversity corridor between the north-eastern and western Indian Ocean [51], such that knowledge of its coral and coral reefs is very important for conservation efforts. This has led to its designation as the world's largest marine protected area [50], [53].

isIn the wake of global climate change and future sea surface warming predicted for this region and others [24], we conducted this study, 1. To document the diversity of *Symbiodinium* in seven common scleractinian species in the Chagos Archipelago and 2. To know whether such remote reefs contribute to some unique *Symbiodinium* clades/types specific to corals present and, if so, discuss the implication of such associations. Under the scenario of temperature and turbidity in determining the regional diversity of *Symbiodinium* [22], we expected proportionately less clade D relative to clade C in our coral samples, as predicted by ecological characteristics of the Chagos where water clarity is high.

## Materials and Methods

### Ethics Statement

Coral tissues were collected and exported from British Indian Ocean Territory with permission granted by the Administrator of the Territory, Foreign and Commonwealth Office, London, United Kingdom.

**Figure 3:**
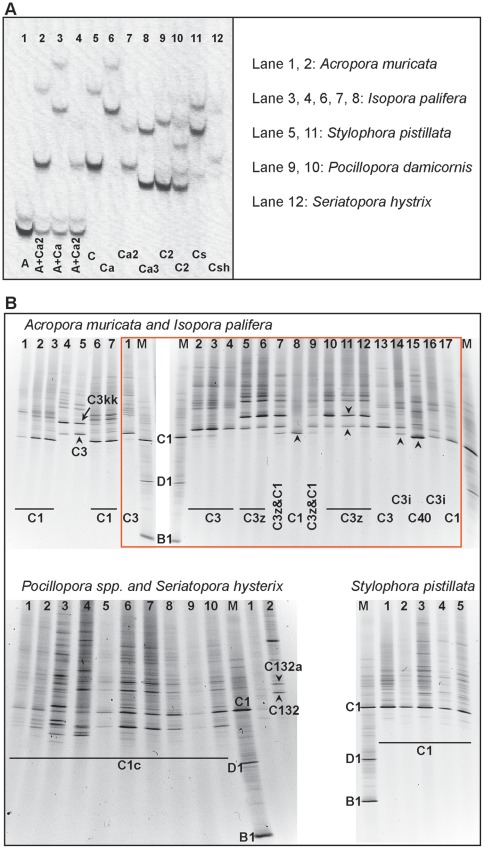
ITS1-SSCP and ITS2-DGGE fingerprints of Symbiodinium types from representative samples of seven corals species from the Chagos. Archipelago. Information of DNA samples from different coral species run in each lane of ITS1-SSCP is shown next to the gel photo (A). For ITS2-DGGE analysis, samples from all representative *Symbiodinium* types are shown on the gel (B). Sample number is shown on each lane gel with markers denoted by the letter M on each gel. *Symbiodinium* type name is shown at the bottom of the lane. *Acropora muricata* – Lane 1–7; *Isopora palifera* – Lane 1–17 (inside red box); *Pocillopora damicornis* – Lane 1–4; *Pocillopora verrucosa* – Lane 5–7; *Pocillopora eudouxi* – Lane 8–10; *Seriatopora hystrix* – Lane 1 and 2 and *Stylophora pistillata* – Lane 1–5. The bands pointed with black arrows were cut and sequenced to confirm the *Symbiodinium* types.

**Figure 4:**
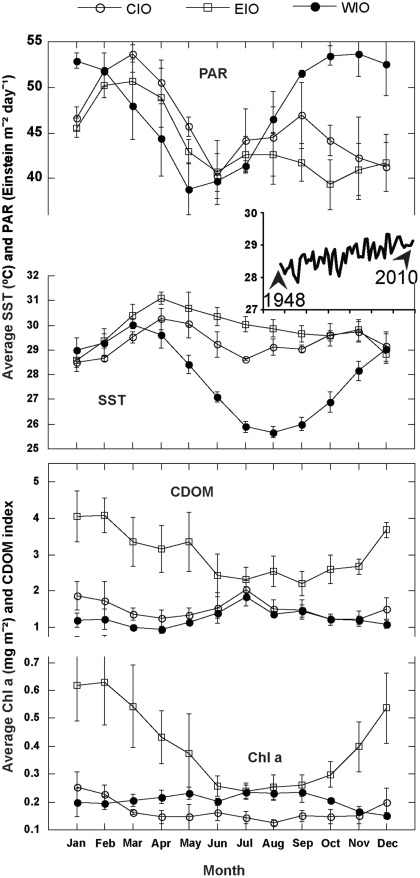
Environmental data from Central, East, and West Indian Ocean. Monthly average photosynthetically active radiation (PAR), sea surface temperature (SST), colored dissolved organic matter (CDOM) and Chlorophyll *a* (Chl *a*) data were obtained online from satellite data banks of NOAA. The sea surface temperature data is between 1948–2010 and PAR, Chl *a* and CDOM data is between 2005–2010.

### Sample sites and field collection

Seven common scleractinian coral species (*Isopora palifera*, *Acropora muricata*, *Pocillopora damicornis*, *P. eydouxi*, *P. verrucosa*, *Stylophora pistillata* and *Seriatopora hystrix*) were sampled at depths of 2 m to 7 m from five parts of the Chagos Archipelago in March 2006 (Fig 1). Sampled sites were Salomon, Peros Banhos and Diego Garcia atolls, and from Eagle Islands and Three Brothers from the Great Chagos Bank. In all 262 samples (Table 1) collected from 7 coral species were preserved in 95% ethanol for DNA extraction.

### Molecular analysis of Symbiodinium clade and types

Use of combinations of genetic analyses better resolves the *Symbiodinium* identity in a given sample [54]. Thus, we used three techniques to analyze *Symbiodinium* clades/types from the extracted DNA. DNA extraction followed the protocol of [55] followed by amplification of DNA using the nuclear ribosomal internal transcribed spacer 1 (ITS1) regions and the nuclear ribosomal internal transcribed spacer 2 (ITS2) regions (see below). Before analyzing the samples using ITS1 and ITS2, DNA from all the samples were screened for *Symbiodinium* clades using large subunit ribosomal DNA (lsur-DNA) restriction fragment length polymorphism (lsurDNA-RFLP) and further confirmed with small subunit ribosomal DNA-RFLP (ssurDNA-RFLP) (Fig. S1). RFLP analysis was carried out following the protocols of [42].

### ITS1 and Single Strand Confirmation Polymorphism (SSCP)

The ITS1 region was amplified using the primer set with a fluorescent Hex-labelled forward primer ‘SymITSFP (5′-CTCAGCTCTGGACGTTGYGTTGG-3′) and ‘SymITSRP’ (5′-TATCGCRCTTCRCTGCGCCCT-3′). Single-stranded conformation polymorphism (SSCP) was performed following the protocol described in [56]. SSCP were preformed using Gelscan 3000 (Corbett Research). PCR products were first denatured at 95^°^C for 3 min and on ice immediately for 3 min. Then PCR products were loaded onto the 4% nondenaturing TBE-polyacrylamide gel (1.5 ml of 40% acrylamide / bis- acrylamide (37.5∶1), 11.445 ml of ddH_2_O, 0.3 ml of 100% glycerol, 0.9 ml 10x TBE, 30 µl of 25% APS and 30 µl of TEMED), pulsed into gel for 25s and flushed. This was followed by a run in 1200 V, 22^°^C with 0.6x TBE buffer for 40 min.

### ITS2 and Denaturing gradient gel electrophoresis (DGGE)

ITS2 region were amplified using primer set ‘ITSintfor2’ (5′-AAT TGC AGA ACT CCG TG-3′) and ‘ITS2 clamp’ (5′-CGC CCG CCG CGC CCC GCG CCC GTC CCG CCG CCC CCG CCC GGG ATC CAT ATG CTT AAG TTC AGC GGG T-3′) from [57] and using touch-down PCR [27]. PCR products of ITS2 were electrophoresed using 45–80% denaturing gradient gels for 16 h on CBS Scientific system (Del Mar, CA, USA). Gels were stained with 1× SYBR Gold (Life Technologies, Invitrogen, USA) for 20 min, and were photographed using a gel documentation system (Vilber Lourmat, France).

The method used for assigning the ITS2-DGGE fingerprint followed [22]. Prominent bands of each fingerprint were sent for direct sequencing then matched with the sequences from Genbank (Table S1).

### Direct sequencing

For samples of Acroporidae for which ITS2-DGGE did not successfully detect the presence of clade A (this might be due to the low copy number of clade A), the region between ITS1 (primer SymITSFP) to lsurDNA (primer D1/D2 R) was amplified, cloned and three clones for each sample were sequenced. The molecular cloning and sequencing methods were followed as per [55].

### Environmental Parameters

Monthly mean SST values (1948–2010) were acquired from NOAA Earth System Research Laboratory NCEP/NCAR data (http://www.esrl. noaa.gov/psd/cgi-bin/data/timeseries/timeseries1. pl; downloaded 14 Mar. 2010), and chlorophyll *a* and coloured dissolved organic matter (CDOM) (2005-2010) records were acquired from the Giovanni online data system (http://gdata1.sci.gsfc.nasa.gov/ daac-bin/G3/gui.cgi? instance_id = ocean_month; downloaded 14 Mar. 2010), which was developed and is maintained by the NASA Goddard Earth Sciences (GES) Data and Information Services Center (DISC), MD, USA. All the data were acquired from 3 regions of the Indian Ocean; Central Indian Ocean (CIO) – Chagos Archipelago; West Indian Ocean (WIO) – Zanzibar; and East Indian Ocean (EIO) – Andaman Sea.

## Results

*Symbiodinium* nomenclature depends on the method of molecular analysis used. For example, types C and Ca in SSCP is designated as C1 and C3 in DGGE. Since we have utilized both SSCP and DGGE for analysis, to avoid confusion, throughout the text we will follow the nomenclature used for DGGE (see Table 2 for more details).

### PCR-SSCP of ITS1

Separate taxonomic units were identified within clade C; C1, C3, C3z, C40, Ca5, C1c, C_2_, Cs and Csh. SSCP results for *A. muricata*, *P. damicornis, P. eydouxi, P. verrucosa, S. pistillata and S. hystrix are shown in* *Fig. 3A* *. Symbiodinium* type diversity in *Pocillopora damicornis*, *P. eydouxi*, *P. verrucosa*, *S. pistillata* and *S. hystrix* revealed the presence of C1c and C_2_ in three *Pocillopora* species, Cs in *S*. *pistillata* and *S. hystrix*, and Csh in *S. hystrix* (Fig. 3A). The coral *I. Palifera* was found to be associated with the greatest number of symbionts including 5 taxa from clade C (C3, C3z, Ca5 and C3z, and C3i; Fig. 3A) and with *Symbiodinium* type A1 within clade A (hereafter referred to as *Symbiodinium* A1). For *A*cropora *muricata*, clade C diversity was less, with only C3i and C3z types, occurring with *Symbiodinium* A1. C3z in *A. muricata* was always found along with *Symbiodinium* A1 (Fig. 3A).

### PCR-DGGE of ITS2

Results of ITS2 analysis also showed *Symbiodinium* clade C: C1, C1c, C3, C3i, C3z, C3kk, C132a, C132 and C40 (Fig. 3B). Specific types of C found in Acroporidae were C1, C3, C3z and C40. In *Pocillopora* spp, DGGE analysis showed the presence of C1c (Fig. 3B) and C_2_, while in *S. pistillata* and *S. hystrix* this method revealed the presence of C1 together with an unknown type Cs. The results also showed the presence of two novel ITS2 types, C132a and C132 in *S. hystrix* (Fig. 3B)

Although clade A was not detected by DGGE (this might be due to low copy number of clade A), results from SSCP, lsurDNA and ssurDNA-RFLP showed the presence of clade A in *Acropora* species. Analysis of ITS2 sequences further indicated that the Clade A in *Isopora* and *Acropora* from Chagos corresponds to *Symbiodinium* A1 from other regions in the Indian Ocean and the Pacific (Fig. S2) [22], [46].

## Discussion

Several studies in the past few years have advanced our knowledge on the biogeographic distribution of *Symbiodnium* diversity in corals and have contributed to our understanding of the coral-*Symbiodinium* association with respect to their specificity to regional environments [17]–[22]. Notably, [22] compared symbiosis patterns in the Indian Ocean (north-east and west Indian Ocean) with the Great Barrier Reef. This showed the occurrence of high incidences of clade D *Symbiodinium* in coral hosts in high temperature, turbid waters of the Andaman Sea (Thailand) compared with clade C in the Great Barrier Reef and Zanzibar. It was suggested that high temperature and turbidity might explain, in part, the ecological success of clade D in these areas [22].

Samples form the Chagos that we analyzed did not show any presence of clade D, even though the composition of *Symbiodinium* varied among reefs (Fig. 2). The absence of clade D in this survey could be because insufficient taxa were sampled. However, not one of the 262 specimens of seven species of major and abundant coral species in the Chagos showed the presence of *Symbiodinium* clade D. Our results indicate high incidence of *Symbiodinium* clade C types with occasional occurrence of *Symbiodinium* A1 in the samples analyzed, which is different form the previously investigated locations in the Indian Ocean.

The type of coral-*Symbiodinium* association in the Chagos observed through this study may be due to the fact that the central Indian Ocean atolls are bathed by water very low in dissolved organic matter (CDOM) with much less pelagic chlorophyll than the northeast Indian Ocean (Andaman Sea) and west Indian Ocean (Zanzibar) (Fig. 4 see also [22], [58]). Water quality not only affects the distribution of corals but also the *Symbiodinium* composition [22], [59]. Also, the mass mortality in 1998 in the Chagos archipelago was triggered as least as much by high irradiance as by warming of seawater; the trade winds that year did not develop [60] leading to prolonged calm sea surface conditions conducive to greater light penetration [61]. This is illustrated by many examples of coral colonies whose shaded sides survived while top surfaces exposed to light did not [62]. The bleaching in the central Indian Ocean in recent years appears to be as much a consequence of very stable water layers and thermoclines given extended periods of low winds, leading to enhanced light penetration, as it was to raised temperatures [61], [63]. However, the average sea surface temperatures in the Chagos atolls are around 28°C, with the warmest being about 30°C [64], which is cooler than in the north-east Indian Ocean (Andaman Sea) where average sea surface temperatures exceed 31°C in summer (Fig. 4). All the above-described conditions can be conducive to the presence of *Symbiodinum* clade C and A in the corals of the Chagos Archipelago. But these correlations needs to be explored with caution since presence of corals and their association with a particular *Symbiodnium* types dose not necessarily correlate with their physiological adaptation to the prevailing environmental conditions in a given area.

### Symbiodinium type diversity

Previous studies have shown that it is important to resolve the high diversity within *Symbiodinium* clade C [20], [22], [34], [46], [65]. *Symbiodinium* types C1 and C3 are common in the Pacific Ocean and Indian Ocean [20], [22]. In this study we could detect at least 14 types of clade C; C1, C1c, C3, C3z, C3i, C40, C3kk, C132a, C132, C40, Ca5, C_2_, Cs and Csh. *I. palifera* had most diverse *Symbiodinium* clade C types (C3, C3z, C3i, C40 and Ca5) (Fig. 3B). The variety of C types present in *I. palifera* may be due to vertical transmission of *Symbiodinium* from parent corals [19]. *A. muricata* hosted type C1 and C3, C3kk (novel *Symbiodinium* type) together with *Symbiodinium* A1 (29% of total samples analyzed), otherwise it was mainly associated with C1. The Presence of *Symbiodinium* A1 in *Acroporidae* in the Chagos is consistent with previous reports of these associations at various locations in the Pacific [46]. Similarly, two *Symbiodinium* types C40 and C3z found in the Acroporidae have been previously recorded in the west Pacific Ocean, eastern Indian Ocean off the coast of Australia and at several locations in Thailand respectively [19], [22], [66]. This indicates that *Acroporidae* may be flexible in its association with different *Symbiodinium* clades, however, whether such flexibility is related to local environmental conditions in the Chagos is a matter of future investigations. *Symbiodinium* type Cs was associated with both *S*. *hystrix* and *S*. *pistillata,* whereas Csh, C132a, C132 were only found in *S*. *hystrix* (Fig. 3B). It has been observed that association of C1b-c in *Pocillopora* species is generally in the clear water reefs in the West Pacific Ocean [59]. This might explain the presence of type C1c (close related with C1b-c) in *Pocillopora* spp in the Chagos archipelago (Fig. 3B). C1c is a common *Symbiodinium* type that has been found in Pocilloridae in the Pacific [19]. However, we could not detect the presence of *Symbiodinium* type C1h that is associated with *Pocillipora* spp. in Zanzibar [22].

### What makes Symbiodinium clade C dominant in the corals of the Chagos Archipelago?

Patterns of *Symbiodinium* associated with corals sampled from Chagos can be explained by the different environmental conditions that prevail in different parts of the Indian Ocean (see Fig. 4). This ocean lacks equatorial upwelling due to the climatological winds, which tend to be westerly rather than easterly. As a result of this, a warm pool is found in the Eastern Indian Ocean rather than in the west, as is the case in the tropical Atlantic and Pacific. This is attributed to the presence of large-scale, land-sea contrasts between the Asian land mass and the Indian Ocean, resulting in the monsoonal winds and seasonal reversing of currents north of ∼20°S. Also lack of winds over the surface of the ocean observed in the central Indian Ocean results in less chlorophyll and CDOM (Fig. 4). The average sea surface temperature in the Chagos is usually <30°C (Fig. 4, also see [67]) with temperatures exceeding 31°C on rare occasions, together with high PAR that results in coral bleaching [62], [63]. This is conducive to the dominance of *Symbiodinium* clade C and presence of clade A to a certain extent in the Chagos corals. However, changing environmental conditions as a result of global climate change (see Fig. 4) shows an increasing trend in average sea surface temperature, which might also be working against such associations.

### Conclusion

Time will reveal whether corals in the Chagos Archipelago adapt to the global climate change and increase in average sea surface temperatures. Presently there is no sign of high temperature resistant *Symbiodinim* clades (clade D) in the corals of the Chagos that we analyzed. The marine environment of the Chagos Archipelago is an exceptional place, which is a result of the absence of overfishing, pollution and minimal other human impacts. The reefs of the Chagos make up perhaps 50% of the total reef area in the Indian Ocean that remains in the least disturbed, low threat category. The view that reefs in the Chagos need strong protection is based not only on studies of the Chagos itself, but from knowledge and observation of the generally poor condition of reefs elsewhere in the Indian Ocean (from Madagascar to Kuwait, and from Sri Lanka westwards) and other oceans too. Now, since the Chagos Archipelago has been designated as a no-take MPA, it is suggested that more such investigations need to be undertaken to assess the overall situation of *Symbiodinium* diversity and distribution in corals of the Chagos, which would facilitate better understanding of the robustness of the corals in this archipelago and help in better management of this large marine protected area in the central Indian Ocean.

### Future directions

Our study has shown that most common coral species in the Chagos archipelago are associated with *Symbiodinium* clade C and A. Whether such coral-*Symbiodinium* associations present in the Chagos are physiologically adapted or acclimated to environmental conditions prevailing in the Chagos Archipelago and environmental tolerances of these *Symbiodinium* types needs to be confirmed through laboratory based physiological experiments.

## Supporting Information

Figure S1 **RFLP (28S and 18S) pattern of Symbiodinium clades in corals from Chagos Archipelago.** RFLP banding profiles shown for 28S rDNA (a) include: type A (lane 2), type A+C (lanes 3 and 4), type C+Csh (Csh; lane 5), type C+Cs (Cs; lanes 6 and 7) and type C (lanes 8–11), with the 100 bp DNA marker in lane 1. RFLP patterns of 18S rDNA (b) include: type A (lane 2), type A+C (lane 3), type C (lanes 4, 6 and 7) and type C2 (lane 8). The 100 bp DNA marker is in lane 1.
(EPS)

Figure S2 **Phylogenetic analysis of 28S-rDNA sequences.** Maximum likelihood tree of all the samples from this study combined (a) and Neighbour-joining trees showing details of Clade C group (b) and clade A group (c). Samples from this study are represented by colored letters.
(EPS)

Table S1 **The biogeographic and host information for the Genbank Accession numbers of Symbiodinium used for phylogenetic analysis.**
(DOC)
