# Supplementary material for: Diversity and Distribution of Symbiodinium Associated with Seven Common Coral Species in the Chagos Archipelago, Central Indian Ocean
Source: PLoS One. 2012 May 2;7(5):e35836. doi: 10.1371/journal.pone.0035836 (PMC3342320; doi:10.1371/journal.pone.0035836)
Supplement: Table S1 — The biogeographic and host information for the Genbank Accession numbers of Symbiodinium used for phylogenetic analysis. (DOC) [file pone.0035836.s003.doc]

| **Host species** | **Location** | **Accession Number** | **Clade** |
| --- | --- | --- | --- |
| ***Tridacna gigas*** | Indo-pacific | AF060897 | A |
| ***Acropora longicyathus*** | GBR | AF349556 | A |
| ***Acropora longicyathus*** | GBR | AF349557 | A |
| ***Porites nigrecens*** | Reunion | AJ308903 | A |
| ***Porites porites*** | Bermuda | AY074968 | A |
| **Firecoral** | GBR | AY239388 | A |
| ***Acropora valida*** | Mombasa | AY588453 | A |
| ***Acropora cervicornis*** | Caribbean | U63480 | A |
| ***Montastraea annularis*** | Panama | AY074966 | B |
| ***Plesiastrea versipora*** | GBR | AF170144 | B |
| ***Seriatopora hystrix*** | Okinawa | AF349544 | C |
| ***Seriatopora hystrix*** | GBR | AF349552 | C |
| ***Montipora verrucosa*** | Hawaii | AF427462 | C |
| ***Stylophora pistellata*** | Penghu | AY139254 | C |
| ***Pocillopora damicornis*** | Penghu | AY139257 | C |
| ***Acropora muricata*** | Penghu | AY139260 | C |
| ***Montipora* sp.** | GBR | AY239383 | C |
| ***Acropora* sp.** | GBR | AY239386 | C |
| ***Acropora hyacinthus*** | Kisite | AY588434 | C |
| ***Acropora valida*** | Mombasa | AY588451 | C |
| ***Pocillopora damicornis*** | Malindi | AY588460 | C |
| ***Pocillopora damicornis*** | Malindi | AY588461 | C |
| ***Pocillopora damicornis*** | Mombasa | AY588463 | C |
| ***Acropora cervicornis*** | Caribbean | U63481 | C |
| ***Seriatopora hystrix*** | Palau | AF349546 | D |
| ***Acropora palifera*** | Kenting | AY139230 | D |
